# Influence of Plasmodium falciparum Calcium-Dependent Protein Kinase 5 (PfCDPK5) on the Late Schizont Stage Phosphoproteome

**DOI:** 10.1128/mSphere.00921-19

**Published:** 2020-01-08

**Authors:** Karin Blomqvist, Michaela Helmel, Chengqi Wang, Sabrina Absalon, Tetanya Labunska, Rachel M. Rudlaff, Swamy Adapa, Rays Jiang, Hanno Steen, Jeffrey D. Dvorin

**Affiliations:** a Division of Infectious Diseases, Boston Children’s Hospital, Boston, Massachusetts, USA; b Department of Pediatrics, Harvard Medical School, Boston, Massachusetts, USA; c Department of Microbiology, Harvard Medical School, Boston, Massachusetts, USA; d Department of Microbiology, Tumor and Cell Biology, Karolinska Institutet, Stockholm, Sweden; e Department of Pathology, Boston Children’s Hospital, Boston, Massachusetts, USA; f Department of Pathology, Harvard Medical School, Boston, Massachusetts, USA; g Center for Global Health & Infectious Diseases Research, Department of Global Health, College of Public Health, University of South Florida, Tampa, Florida, USA; Johns Hopkins Bloomberg School of Public Health

**Keywords:** *Plasmodium falciparum*, calcium-dependent protein kinase, malaria, phosphoproteome

## Abstract

Protein kinases are important mediators of signal transduction in cellular pathways, and calcium-dependent protein kinases (CDPKs) compose a unique class of calcium-dependent kinases present in plants and apicomplexans, including *Plasmodium* parasites, the causative agents of malaria. During the asexual stage of infection, the human malaria parasite Plasmodium falciparum grows inside red blood cells, and P. falciparum calcium-dependent protein kinase 5 (PfCDPK5) is required for egress from the host cell. In this paper, we characterize the late-schizont-stage P. falciparum phosphoproteome by performing large-scale phosphoproteomic profiling on tightly synchronized parasites just prior to egress, identifying 2,704 phosphorylation sites on 919 proteins. Using a conditional knockdown of PfCDPK5, we identify 58 phosphorylation sites on 50 proteins with significant reduction in levels of PfCDPK5-deficient parasites. Furthermore, gene ontology analysis of the identified proteins reveals enrichment in transmembrane- and membrane-associated proteins and in proteins associated with transport activity. Among the identified proteins is PfNPT1, a member of the apicomplexan-specific novel putative transporter (NPT) family of proteins. We show that PfNPT1 is a potential substrate of PfCDPK5 and that PfNPT1 localizes to the parasite plasma membrane. Importantly, P. falciparum egress relies on many proteins unique to Apicomplexa that are therefore attractive targets for antimalarial therapeutics.

**IMPORTANCE** The malaria parasite Plasmodium falciparum is a major cause of morbidity and mortality globally. The P. falciparum parasite proliferates inside red blood cells during the blood stage of infection, and egress from the red blood cell is critical for parasite survival. P. falciparum calcium-dependent protein kinase 5 (PfCDPK5) is essential for egress; parasites deficient in PfCDPK5 remain trapped inside their host cells. We have used a label-free quantitative mass spectrometry approach to identify the phosphoproteome of schizont-stage parasites just prior to egress and identify 50 proteins that display a significant reduction in phosphorylation in PfCDPK5-deficient parasites. We show that a member of the Apicomplexan-specific transport protein family, PfNPT1 is a potential substrate of PfCDPK5 and is localized to the parasite plasma membrane. P. falciparum egress requires several proteins not present in human cells, thus making this pathway an ideal target for new therapeutics.

## Abstract (precis)

The malaria parasite Plasmodium falciparum is a major cause of morbidity and mortality globally. The P. falciparum parasite proliferates inside red blood cells during the blood stage of infection, and egress from the red blood cell is critical for parasite survival. P. falciparum calcium-dependent protein kinase 5 (PfCDPK5) is essential for egress; parasites deficient in PfCDPK5 remain trapped inside their host cells. We have used a label-free quantitative mass spectrometry approach to identify the phosphoproteome of schizont-stage parasites just prior to egress and identify 50 proteins that display a significant reduction in phosphorylation in PfCDPK5-deficient parasites. We show that a member of the Apicomplexan-specific transport protein family, PfNPT1 is a potential substrate of PfCDPK5 and is localized to the parasite plasma membrane. P. falciparum egress requires several proteins not present in human cells, thus making this pathway an ideal target for new therapeutics.

## OBSERVATION

Plasmodium falciparum is the deadliest of the *Plasmodium* species, causing 435,000 deaths in 2017 (1). *Plasmodium* parasites have a complex multihost life cycle requiring both the mosquito and human host for completion. During the human blood stage, the parasite invades erythrocytes and resides within a parasitophorous vacuole where it progresses from early ring to late-schizont-stage parasites. Egress from the infected red blood cell is critical for parasite survival and proliferation, and this process is regulated, in part, by protein phosphorylation (2–5). The molecular events preceding egress are incompletely understood, but two kinases are essential: P. falciparum calcium-dependent protein kinase 5 (PfCDPK5) and a P. falciparum cGMP-dependent protein kinase (PfPKG) (6–8). A protease cascade is also essential for egress, involving the serine protease PfSUB1-mediated cleavage of multiple substrates, including PfMSP1, PfSERA5, and PfSERA6 (9).

PfCDPK5 deficiency results in fully mature parasites that are trapped inside their host cells (6). PfCDPK5 has a dynamic localization; initially, the kinase colocalizes with apical merozoite organelles called micronemes, then fills the apical region of the merozoites, and finally localizes diffusely near the parasite plasma membrane prior to or during egress (8). It is required for microneme discharge and cooperates with PfPKG to fully activate the protease cascade (8). The interacting proteins of PfCDPK5 and its substrates remain unknown.

In the current study, we extended the knowledge of PfCDPK5 interacting proteins and the schizont phosphoproteome by performing large-scale phosphoproteomic profiling on late-stage (48 h postinvasion [h p.i.]) schizonts. In five comprehensive parasite lysates from tightly synchronized late-stage schizonts, we identified a total of 2,704 phosphorylation sites on 919 proteins. Using a conditional knockdown of PfCDPK5, we show that 58 phosphorylation sites on 50 proteins displayed significant reduction in phosphorylation upon PfCDPK5 knockdown. Gene Ontology (GO term) enrichment analysis reveals that transmembrane- and membrane-associated proteins and proteins associated with transport activity are significantly enriched in the PfCDPK5-specific phosphoproteome. Among the upregulated phosphoproteins is PfNPT1 (PF3D7_0104800), a member of the apicomplexan-specific novel putative transporter (NPT) family of proteins. We show that PfNPT1 is a substrate of PfCDPK5 *in vitro* and colocalizes with PfCDPK5 at the parasite plasma membrane.

To identify the influence of PfCDPK5 on the schizont-stage phosphoproteome, we utilized parasites with a conditional knockdown of PfCDPK5, where fusion with a destabilizing domain (DD) allows inducible protein regulation via rapid degradation of the fusion in the absence of the stabilizing ligand Shield-1 (Shld1) (6, 10). Parasites were tightly synchronized by Percoll isolation of schizonts followed by sorbitol selection of newly invaded rings after 4 h. Sorbitol selection was repeated in the following cycle, and parasites were then maintained with or without Shld1. At 42 h postinvasion (h p.i.), the parasites were treated with the cysteine protease inhibitor E64, arresting parasites prior to rupture of the erythrocyte membrane (9). Parasites were subsequently harvested at 48 h p.i. After trypsin digestion and gel-free desalting, phosphopeptides were enriched using immobilized metal affinity chromatography (IMAC) before label-free liquid chromatography-tandem mass spectrometry (LC-MS/MS) was performed. Three biological replicates were performed, the last replicate in technical triplicate, resulting in a total in five data sets.

## 

### The phosphoproteome of the mature P. falciparum schizont and the influence of PfCDPK5.

**FIG 1:**
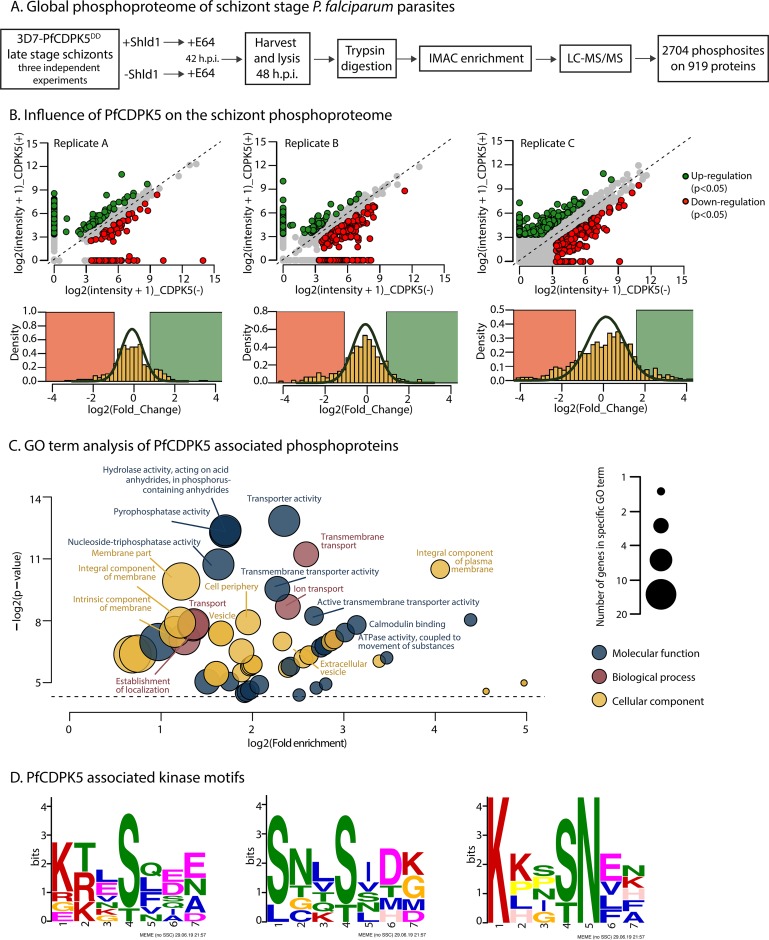
(A) Global phosphoproteome of P. falciparum. An outline of the experimental phosphoproteomic workflow is presented. For the PfCDPK5-depleted condition, Shld1 was removed at the early ring stage. In order to arrest the parasites at the mature schizont stage, protease inhibitor E64 was added to the cultures at 42 h p.i. The parasite cultures were harvested at 48 h p.i., and the red blood cells were lysed using saponin. The released parasites were subsequently lysed by the use of urea and sonication. Protein samples were digested with trypsin, and phosphoproteins were enriched using IMAC. LC-MS/MS was performed, and data analysis was carried out using Maxquant software with a false-discovery rate (FDR) of 1%. Protein preparation was performed in three biological replicates, the third experiment was done in technical triplicate. (B) Influence of PfCDPK5 on the schizont phosphoproteome. Replicate A, Replicate B, and Replicate C correspond to the three biological replicates. (Upper panel) Scatter plots showing the phosphorylation site intensity distribution in the PfCDPK5 [+] Shld1 samples compared to the [-] Shld1 samples. The red and green circles indicate significantly (*P* < 0.05) differentially phosphorylated residues. (Lower panel) Histogram showing the (log2) fold change of phosphorylation site intensity between the PfCDPK5 [+] Shld1 and [-] Shld1 samples. The dark green curve indicates Gaussian distribution fitting by the maximum log likelihood method. Phosphopeptides with fold changes in the top 10% and bottom 10% were removed for Gaussian fitting. (C) GO term analysis of PfCDPK5-associated phosphoproteins. The Gene Ontology (GO) enrichment analysis was performed using PlasmoDB for phosphoproteins that were significantly enriched in the [+] Shld1 samples. The full list with identification of all enriched terms is provided in Data Set S5. (D) PfCDPK5-associated kinase motifs. The predominant phosphorylation motifs are shown for the phosphorylation sites that were significantly enriched in the [+] Shld samples identified using MEME software.

We identified the global phosphoproteome of P. falciparum schizonts at 48 h p.i., including all detected phosphorylation sites in all five data sets. In total, we identified 2,704 phosphorylation sites from 919 proteins (Fig. 1A; see also Data Set S1 to S3 in the supplemental material). To identify differentially phosphorylated proteins in PfCDPK5-deficient parasites, we first used stringent criteria to exclude ribosomal proteins, transcription factors, proteins with predominantly nuclear localization, proteins not conserved in all *Plasmodium* species, and proteins showing variant levels of expression (e.g., PfEMP1, Rifins, Stevors). Further bioinformatic analysis identified 58 phosphorylation sites on 50 proteins with a significant reduction in phosphorylation in the PfCDPK5-deficient parasites in at least two of three biological replicates with *P* values of <0.05 (shown in Fig. 1B; see also Data Set S4, sheet 1).

10.1128/mSphere.00921-19.2 DATA SET S1 Raw intensity data from all three (five, including technical replicates) LC-MS/MS replicates. Download Data Set S1, XLSX file, 0.5 MB. Copyright © 2020 Blomqvist et al. 2020 Blomqvist et al. This content is distributed under the terms of the Creative Commons Attribution 4.0 International license.

10.1128/mSphere.00921-19.3 DATA SET S2 Sum normalized intensity data for the three replicates, including mean intensity data for replicate C (done in technical triplicate). Download Data Set S2, XLSX file, 0.3 MB. Copyright © 2020 Blomqvist et al. 2020 Blomqvist et al. This content is distributed under the terms of the Creative Commons Attribution 4.0 International license.

10.1128/mSphere.00921-19.4 DATA SET S3 Merged phosphopeptides. Data represent grouping of all peptides that had the same phosphorylated residues. The table includes corresponding phosphorylated residues and genes. Download Data Set S3, XLSX file, 0.5 MB. Copyright © 2020 Blomqvist et al. 2020 Blomqvist et al. This content is distributed under the terms of the Creative Commons Attribution 4.0 International license.

10.1128/mSphere.00921-19.5 DATA SET S4 Phosphorylation sites with significantly differential phosphorylation in at least two of three biological replicates with *P* values of <0.05. (Sheet 1) Phosphorylation sites significantly enriched in the PfCDPK5-repleted parasites. (Sheet 2) Phosphorylation sites significantly enriched in the PfCDPK5-depleted parasites. Download Data Set S4, XLSX file, 0.02 MB. Copyright © 2020 Blomqvist et al. 2020 Blomqvist et al. This content is distributed under the terms of the Creative Commons Attribution 4.0 International license.

Gene Ontology (GO term) enrichment analysis revealed that transmembrane- and plasma membrane-associated proteins as well as proteins associated with transport activity and transmembrane transport activity were statistically significantly enriched (*P* < 0.05) in the PfCDPK5-specific phosphoproteome (Fig. 1C; see also Data Set S5).

10.1128/mSphere.00921-19.6 DATA SET S5 Complete list of significant Gene Ontology (GO) terms for the phosphoproteins that are enriched in the CDPK5-replete ([+] Shld1) samples. Download Data Set S5, XLSX file, 0.02 MB. Copyright © 2020 Blomqvist et al. 2020 Blomqvist et al. This content is distributed under the terms of the Creative Commons Attribution 4.0 International license.

### The PfCDPK5-associated kinase motifs.

Using the MEME algorithm software (11), we identified three phosphorylation site motifs that were significantly associated with PfCDPK5 phosphorylation (Fig. 1D). This analysis revealed that PfCDPK5 has a preference for motifs with basic residues, e.g., arginine and lysine at the *P*^−2^ and *P*^−3^ positions. These findings are consistent with earlier studies on PfPKG, PfPKA, and PfCDPK1 where arginine/lysine were found to precede the phosphorylation site in consensus kinase motifs (3, 12, 13).

### PfNPT1 is a PfCDPK5 substrate *in vitro*.

**FIG 2:**
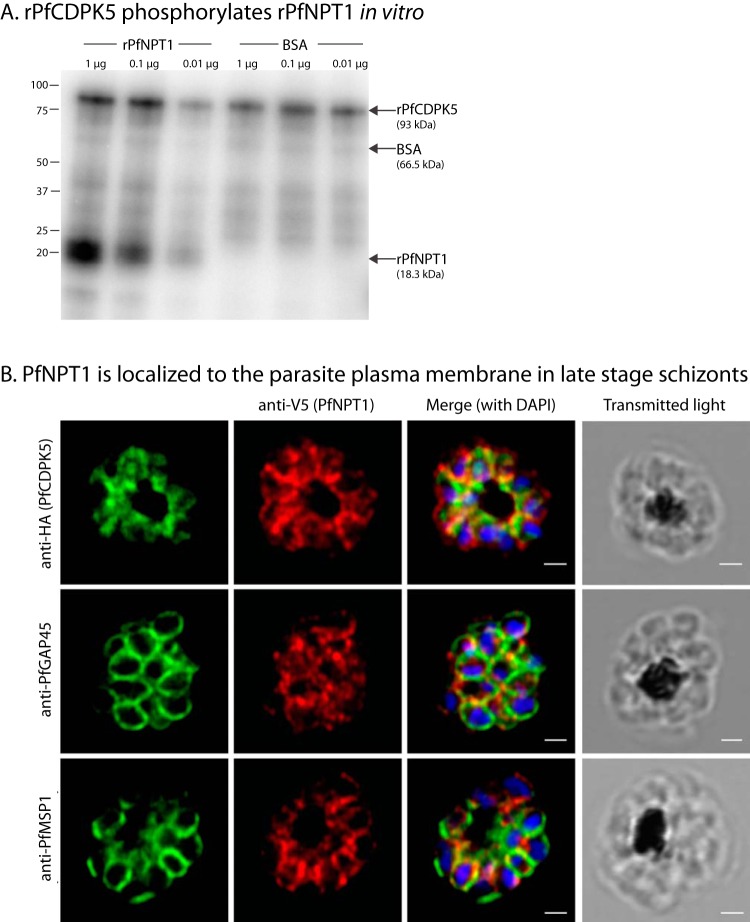
(A) rPfNPT1 is phosphorylated by rPfCDPK5 *in vitro*. *In vitro* kinase reactions were performed with [^32^P]ATP using recombinant versions of PfNPT1 and PfCDPK5. BSA was used as a negative control. (B) PfNPT1 localizes to the parasite plasma membrane. Schizont stage parasites [+] Shld1 were treated with E64, fixed, and probed with anti-HA (PfCDPK5), anti-V5 (PfNPT1), anti-PfGAP45, and/or anti-PfMSP1 antibodies. Nuclei were stained with DAPI (4′,6-diamidino-2-phenylindole). Scale bar, 1 μm.

We identified PfNPT1 (PF3D7_0104800), a member of the apicomplexan-specific novel putative transporter (NPT) family of proteins, as the first protein in the PfCDPK5-specific phosphoproteome. In Toxoplasma gondii, T. gondii NPT1 (TgNPT1) is a selective arginine transporter that is essential for parasite survival (14). In the rodent malaria parasite P. berghei, P. berghei NPT1 (PbNPT1) is essential for the sexual gametocyte stages (15). We found that residue Ser259 in PfNPT1 was significantly differentially phosphorylated (Data Set S4, sheet 1). We recombinantly expressed the intracellular domain (amino acids [aa] 234 to 378) of PfNPT1 (rPfNPT1) and performed *in vitro* kinase assays. The results confirmed that rPfCDPK5 phosphorylates rPfNPT1 *in vitro* (Fig. 2A). Mass spectrometry analysis further confirmed that rPfCDPK5 phosphorylated rPfNPT1 at the identified region but that it did so at the neighboring Ser261 residue. We note that other serine and threonine residues in rPfNPT1 were phosphorylated in addition to the identified region.

### PfNPT1 is localized to the parasite plasma membrane in late-stage schizonts.

We expressed PfNPT1 episomally with a spaghetti monster V5 (smV5) tag (16) in PfCDPK5-3HA-DD parasites (see Fig. S1 in the supplemental material). As demonstrated previously (8), an immunofluorescence assay (IFA) performed by superresolution microscopy on E64-arrested (postegress) parasites showed that PfCDPK5-3HA localization both shows diffusion within the parasite cytoplasm and displays increased signal near the parasite plasma membrane (Fig. 2B). Costaining with anti-V5 and antihemagglutinin (anti-HA) antibodies revealed a partial overlap PfCDPK5 and PfNPT1, particularly in areas near the parasite plasma membrane (Fig. 2B, top row). PfGAP45 is a protein associated with the inner membrane complex which underlies the plasma membrane. Costaining with anti-PfGAP45 and anti-V5 showed partial overlap (Fig. 2B, middle row). However, with antibodies to the parasite plasma membrane protein PfMSP1, we detected strong colocalization of PfNPT1 and PfMSP1 (Fig. 2B, bottom row), demonstrating that PfNTP1 localizes to the parasite plasma membrane. This result agrees with the reported localization of PbNTP1 in P. berghei (15).

10.1128/mSphere.00921-19.1 FIG S1 PfNPT-smV5 plasmid map for episomal expression vector. The full-length coding region for PfNPT1 (PF3D7_0104800) was cloned downstream of the PF3D7_1412100 5′ untranscribed region (5′UTR). The resulting protein represents a fusion between PfNPT1 and the spaghetti monster V5 epitope tag (smV5). The positive selection cassette expresses the dihydroorate dehydrogenase protein from Saccharomyces cerevisiae (ScDHODH). Download FIG S1, PDF file, 1.3 MB. Copyright © 2020 Blomqvist et al. 2020 Blomqvist et al. This content is distributed under the terms of the Creative Commons Attribution 4.0 International license.

### Conclusions.

PfCDPK5 is critical for egress, and PfCDPK5-deficient parasites fail to complete this process, remaining trapped inside their host cell. In this paper, we present a new schizont-stage (48 h p.i.) phosphoproteome from highly synchronized parasites just prior to egress, identifying 2,704 phosphorylation sites from 919 proteins. We used a label-free LC-MS/MS approach which allowed minimal manipulation of proteins and a high level of quantitative proteomic coverage (17). Comparing our results to existing data regarding P. falciparum phosphoproteomes, earlier studies published on P. falciparum schizont stages had reported between 1,177 to 8,463 phosphorylation sites on 650 to 1,673 proteins (2–5, 18, 19). The variation and limited overlap in data from different phosphoproteomic studies are in part due to the variations in the methods used for phosphopeptide enrichment and for LC-MS/MS setup. The use of different time points in the analyses of schizonts included in the different studies contributed to the broad variation in the published P. falciparum schizont phosphoproteomes, with Pease et al. and Solyakov et al. focusing on earlier (36 ± 4 h p.i.) schizonts (4, 5) whereas the study by Treeck et al. included parasites from a broader schizont time window of 40 ± 8 h p.i. (2).

Importantly, we identified 50 proteins that display a significant reduction in phosphorylation in PfCDPK5-deficient parasites. We show that PfNPT1, a member of the apicomplexan-specific novel putative transporter (NPT) protein family, is a potential substrate of PfCDPK5 and is localized to the parasite plasma membrane. Members of the NPT family have been shown to a play key role in cationic amino acid uptake in T. gondii (14). In the rodent malaria parasite P. berghei, PbNPT1 is essential for gametocyte stages (15) and is a cationic amino acid transporter. The egress process in P. falciparum includes several proteins that are not present in human cells, and small molecules could thus potentially be used to block parasite egress by targeting PfCDPK5 and its substrates.

### P. falciparum strains and culture conditions.

P. falciparum parasites were cultured in O+ erythrocytes at 4% hematocrit (20). The parasites were maintained at 37°C under shaking conditions, and RPMI 1640 culture medium (Sigma) was supplemented with 25 mM HEPES (EMD Biosciences), 0.12% sodium bicarbonate (Sigma), 50 mg/liter hypoxanthine (Sigma), and 0.5% Albumax II (Invitrogen). The destabilizing domain (DD) was utilized to regulate the protein level of PfCDPK5 (PF3D7_1337800) in the 3D7 parasite strain as previously described (6). In the absence of the stabilizing ligand Shld1, PfCDPK5-DD is rapidly degraded. During continuous culture, parasites were grown with Shld1 (250 nM) and the selection drug WR99210 (2.5 nM).

### Preparation of Plasmodium falciparum proteins for mass spectrometry.

3D7-PfCDPK5-DD culture (300 ml) was used for each condition (with or without Shld1). When reinvasion had occurred and the parasites were early rings, Shld1 was removed by washing the cultures three times in RPMI medium. Subsequently, Shld1 was added back to the culture for the [+] Shld1 condition. In order to arrest the parasites at the mature schizont stage, protease inhibitor E64 (Sigma) was added to the cultures at 42 h p.i. Cultures were harvested at 48 h p.i. by centrifugation at 400 × *g* for 10 min. The red blood cells were lysed using 0.05% Saponin (Sigma) for 10 min at 4°C. The parasites were pelleted and washed in ice-cold 1× phosphate-buffered saline (PBS) supplemented with protease and phosphatase inhibitors (Roche) and centrifuged at 1,800 × *g* for 10 min at 4°C until the supernatant was clear. The parasites were lysed using modified urea lysis buffer containing 8 M urea, 25 mM Tris (pH 8), 100 mM NaCl, one tablet of protease inhibitor cocktail (Complete Mini; Roche), and one tablet of phosphatase inhibitor cocktail (Roche) per 10 ml of lysis buffer. Fresh buffer was prepared for each experiment. The parasites were lysed on ice for 30 min using 1 ml urea lysis buffer per pellet. The samples were sonicated three times at 30 s each time on ice with 30% output with a 2-min rest on ice between the sonications. In order to remove debris, the samples were centrifuged at 15,000 × *g* for 10 min at 4°C. The protein concentration was calculated using the bicinchoninic acid (BCA) assay (Pierce). The total protein concentration in the final lysates was between 2 and 4 mg. Protein preparation was performed in three biological replicates (replicates A to C), and the third experiment was done in technical triplicate.

### Sample preparation—phosphoproteomics.

Sample preparation performed as described previously by McDowell et al. (21) was applied to our samples with a few changes and without isobaric labeling. Protein samples (300 μg) were reduced using 3 μl 2 M dithiothreitol for 30 min at 37°C and 450 rpm on the thermomixer. The sample was transferred into a 10-kDa FASP filter (Microcon-10 centrifugal filters; Millipore) and centrifuged at room temperature and 14,000 × *g* for about 30 min (until the liquid in the filter was almost gone). A wash with 200 μl 8 M urea (Sigma)–50 mM ammonium bicarbonate (ABC; Sigma) was carried out in a similar fashion. The sample was alkylated with 100 μl 0.05 M 2-iodoacetamid solution (TCI America) in 8 M urea for 30 min at room temperature at 600 rpm on the thermomixer. After centrifugation of the sample at room temperature and 14,000 × *g* for 20 min, the sample was washed three times with 100 μl 8 M urea. Trypsin digestion was carried out using a 1:50 enzyme-to-protein ratio with modified sequencing-grade trypsin (Promega) by incubation at 37°C for 18 h. The lid from the filter was covered with Parafilm to avoid evaporation. After centrifugation at room temperature at 14,000 × *g* for 8 min, a filter washing step with 50 μl 50 mM ABC solution was carried out followed by a filter washing step with 0.5 M NaCl solution. The pH was adjusted to pH 2 to 3 with formic acid, and the samples were desalted on an Oasis HLB column (Waters) (1 cc). The column was equilibrated first by washing the column 4 times with 1 ml 70% acetonitrile (ACN) (in 0.1% formic acid) and 4 times with 1 ml 0.1% formic acid. The sample was passed through the column three times and washed 8 times with 1 ml 0.1% formic acid. If necessary, samples were combined and evaporated to dryness overnight.

### Phosphopeptide enrichment using IMAC separation.

Phosphopeptide separation was performed with an IMAC column using a method similar to that previously described by Ruprecht et al. (22) with a few changes and optimization steps for our samples. For phosphopeptide enrichment, the ProPac IMAC column was charged with Fe(III) chloride (Fisher Scientific). Therefore, the lines of the chromatography system were flushed with 10 ml high-quality water followed by stripping the column with 60 ml of 50 mM EDTA and rinsing the column with 20 ml of 20 mM formic acid. The column was then charged with 6 ml of 25 mM FeCl_3_–10 mM acetic acid (0.3 ml/min). Finally, the column was washed with 40 ml 20 mM formic acid (0.3 ml/min). The column was recharged after every eighth sample. After preparation of the column, the sample was dissolved in 350 μl buffer A (0.07% trifluoroacetic acid [TFA], 30% ACN) and sonicated for 5 min. After loading of the sample, the following run was performed: buffer A (0.07% TFA, 30% ACN); buffer B (0.5% NH_4_OH) (the collection window was for the first 15 ml); loading/injecting of the sample (0.5 ml; 0.1 ml/min), isocratic flow (buffer A: 100%; 2 ml; 0.3 ml/min), linear gradient (to 55% buffer A; 10 ml, 0.3 ml/min), linear gradient (to 0% buffer A; 1.5 ml; 0.3 ml/min), isocratic flow (buffer A: 0%; 1.5 ml; 0.3 ml/min), and isocratic flow (buffer A: 100%; 15 ml; 0.5 ml/min). The fraction with the phosphopeptides as well as those in the four following tubes were collected and evaporated to dryness. Each fraction was dissolved in100 μl 0.1% formic acid and sonicated for 5 min. After the phosphopeptide fraction was combined with the four following fractions, the sample was desalted with an Oasis HLB column in the manner described in the “Sample preparation” section.

### LC-MS/MS analysis.

The sample was dissolved in 12 μl loading buffer (5% ACN, 5% formic acid) and sonicated for 5 min. Samples were placed in an autosampler linked to a nanoflow high-performance liquid chromatography (HPLC) system (nanoLC 400; Eksigent) and a Q Exactive mass spectrometer. For the separation, a nano-cHiPLC Trap column (ReproSil-Pur C_18_-AQ) (200-μm inner diameter by 0.5-mm length, 3-μm pore size, 120 Å) and a nano-cHiPLC column (ReproSil-Pur C18-AQ) (75-μm inner diameter by 15-cm length, 3-μm pore size, 120 Å) were used. Peptides were eluted with a 60-min linear gradient from 93% buffer A (water with 0.2% formic acid) and 7% buffer B (acetonitrile with 0.2% formic acid) to 68% buffer A. The injection volume was 4 μl. The Q Exactive mass spectrometer was run in positive-ion mode. Full scans were carried out at a resolution of 70 k with an automatic gain control (AGC) target of 3 × 10^6^ ions and a maximum injection time of 120 ms, using a scan range of 350 to 2,000 *m*/*z*. For acquisition fo MS/MS data, a normalized collision energy value of 27 was used. Scans were carried out at a resolution of 35k with an AGC target of 3 × 10^6^ ions and a maximum injection time of 120 ms. The isolation window was set to 2 *m*/*z*. An underfill ratio value of 0.5% was set, and a dynamic exclusion duration of 20 s was applied.

### Phosphoproteomics data analysis.

Data analysis was performed using Maxquant (Version 1.5.2.8) and a database of Plasmodium falciparum (isolate 3D7). Phosphorylation (STY) and oxidation (M) was chosen as variable modification and carbamidomethylation as fixed modification. Finally, the evidence file was used to extract the phosphopeptides. Using the modified sequence column, the average intensity of each modified peptide was calculated (Data Set S1). The phosphopeptides were normalized by the sum intensity of each sample (Data Set S2).

### Bioinformatic analysis.

Phosphopeptides deriving from the same gene were merged if they shared at least 8 amino acids flanking the phosphorylation site. All peptides containing multiple (*n*) phosphorylation sites were converted to *n* sequences, each sequence containing a single phosphorylation site. For instance, ENQT(ph)IS(ph)LA DEKEIQILNDLT(ph)SQK in gene PF3D7_0525800 was converted into three strings for individual analysis as follows: (i) ENQT(ph)ISLADEKEIQILNDLTSQK, (ii) ENQTIS(ph)LADEKEIQILNDLTSQK, and (iii) ENQTISLADEKEIQILNDLT(ph)SQK. Data Set S3 provides information on merged phosphopeptides, corresponding phosphorylation sites, and gene as well as normalized intensity data from the three biological replicates.

### Identification of differentially phosphorylated residues and Gene Ontology (GO) enrichment analysis.

To explore the differences in phosphorylation between PfCDPK5-replete and PfCDPK5-deficient parasites, fold change data corresponding to the phosphorylation intensity were calculated for each phosphorylation site in the three biological replicates. To remove noise fluctuation, the normalized intensity value was required to be >9.037135 (75% quantile) in at least one (PfCDPK5-replete or PfCDPK5-deficient) sample for each replicate. Otherwise, the fold change was assigned to a value of 1. The normal distribution was fitted for log2(fold change) by maximum likelihood estimation (MLE). The *P* value of each log2(fold change) was calculated as the probability of a fitted normal distribution value being higher than the calculated value. Phosphorylation site fold changes with a *P* value of <0.05 in at least two biological replicates were identified as corresponding to a PfCDPK5-associated phosphorylation site. Stringent criteria were applied for the bioinformatic analysis with the following exclusion criteria: proteins with nuclear localization, known exported protein, proteins with variant expression (i.e., PfEMP1, Stevors, and Rifins), and proteins involved in transcription and/or translation. The Gene Ontology (GO) enrichment analysis was performed using PlasmoDB (23).

### Identification of PfCDPK5 substrate phosphorylation motifs.

To identify sequence motifs directly or indirectly associated with PfCDPK5 activity, phosphorylation sites whose intensities were significantly enriched in PfCDPK5-replete parasites were extracted. The flanking sequences (±3 amino acids) of the phosphorylated residues were extracted. If the phosphorylation site was located at the extreme end of peptide (<3 amino acids from the sequence end), the missing sequence information was completed based on the protein sequence related to the peptide (downloaded from PlasmoDB [23]). The enriched motifs were identified using the MEME algorithm tool (11).

### Plasmid generation and immunofluorescence assay (IFA) for PfNPT1.

For the PfNPT1-smV5 parasite episomal expression plasmid, pJR118 (24) was modified to place the stage-specific promoter region from PF3D7_1412100 into SalI/NotI sites (forward primer, ACGCGTCGACAAATGCAGATATATAATATGTGAC; reverse primer, ATTTGCGGCCGCTTTTAGAATCAAGATAAATGAACACTAACTTTAATGT), the coding region of PF3D7_0104800 into NotI/XhoI sites (forward primer, TGAGCGGCCGCATGAGTAACTCAATATTCCATAAG; reverse primer, GTACTCGAGATTCATTGGGAGTTTATCCTTTTCTCC), and the smV5 epitope tag into XhoI/KpnI sites (forward primer, CCGCTCGAGATGGGAAAACCTATACCGAACCCCCTC; reverse primer, CGGGGTACCTTAGGTACTATCCAGTCCCAGCAACGG). This plasmid was further modified to exchange the human dihydrofolate reductase selectable marker with yeast dihydroorotate dehydrogenase into the BamHI/HindIII sites.

Immunofluorescence microscopy was carried out as previously described (8, 25). Briefly, 3D7-PfCDPK5 ^3HA-DD^-PfNPT1^sm-V5^ parasites were maintained with or without Shld and E64 was added at 42 h p.i. Air-dried thin films of late-stage parasites was fixed with 1% paraformaldehyde at approximately 48 h p.i., permeabilized with 0.1% Triton X-100 for 10 min, blocked with 3% bovine serum albumin (BSA) at 4°C, and incubated with primary antibodies at 4°C overnight (rat anti-HA [Roche] at 1:50, mouse anti-V5 [Bio-Rad] at 1:500, rabbit anti-PfGAP45 [provided by Julian Rayner] at 1:5,000, and/or mouse anti-PfMSP1 [provided by Anthony Holder] at 1:500). The cells were washed three times with PBS, incubated with secondary antibodies (Alexa Fluor 488 or 555 at 1:10,000), washed again three times with PBS, and mounted with Vectashield containing 4′,6-diamidino-2-phenylindole (Vector Laboratories). Images were obtained on an Airyscan LSM880 microscope.

### *In vitro* kinase assay for PfCDPK5.

The cytoplasmic domain of PfNPT1 was cloned into NcoI/XhoI in pET28b to generate a C-terminally 6×His-tagged expression plasmid. The coding sequence for the cytoplasmic domain of PfNPT1 was synthesized (IDT DNA) with Escherichia coli codon optimization. The recombinant protein was expressed in E. coli and purified on nickel-nitrilotriacetic acid (Ni-NTA) resin per the manufacturer**’**s directions. A 250-ng volume of recombinant PfCDPK5 (6) and 1 μg, 0.1 μg, or 0.01 μg recombinant PfNPT1 or control protein (bovine serum albumin [NEB]) was incubated with kinase buffer (50 mM Tris-HCl [pH 8.0], 50 mM NaCl, 10 mM MgCl_2_, 1.1 mM CaCl_2_) and 0.5 μl of [γ-^32^P]ATP (PerkinElmer) (10 mCi/ml) for 30 min at 30°C. The reactions were stopped by addition of Laemmli sample buffer and boiling for 5 min. The proteins were separated using 4% to 20% TGX SDS-PAGE, and the wet gel was wrapped in plastic wrap and imaged with a phosphorimager screen.
