# Supplementary material for: Influence of Plasmodium falciparum Calcium-Dependent Protein Kinase 5 (PfCDPK5) on the Late Schizont Stage Phosphoproteome
Source: mSphere. 2020 Jan 8;5(1):e00921-19. doi: 10.1128/mSphere.00921-19 (PMC6952208; doi:10.1128/mSphere.00921-19)
Supplement: FIG S1 [file mSphere.00921-19-sf001.pdf]

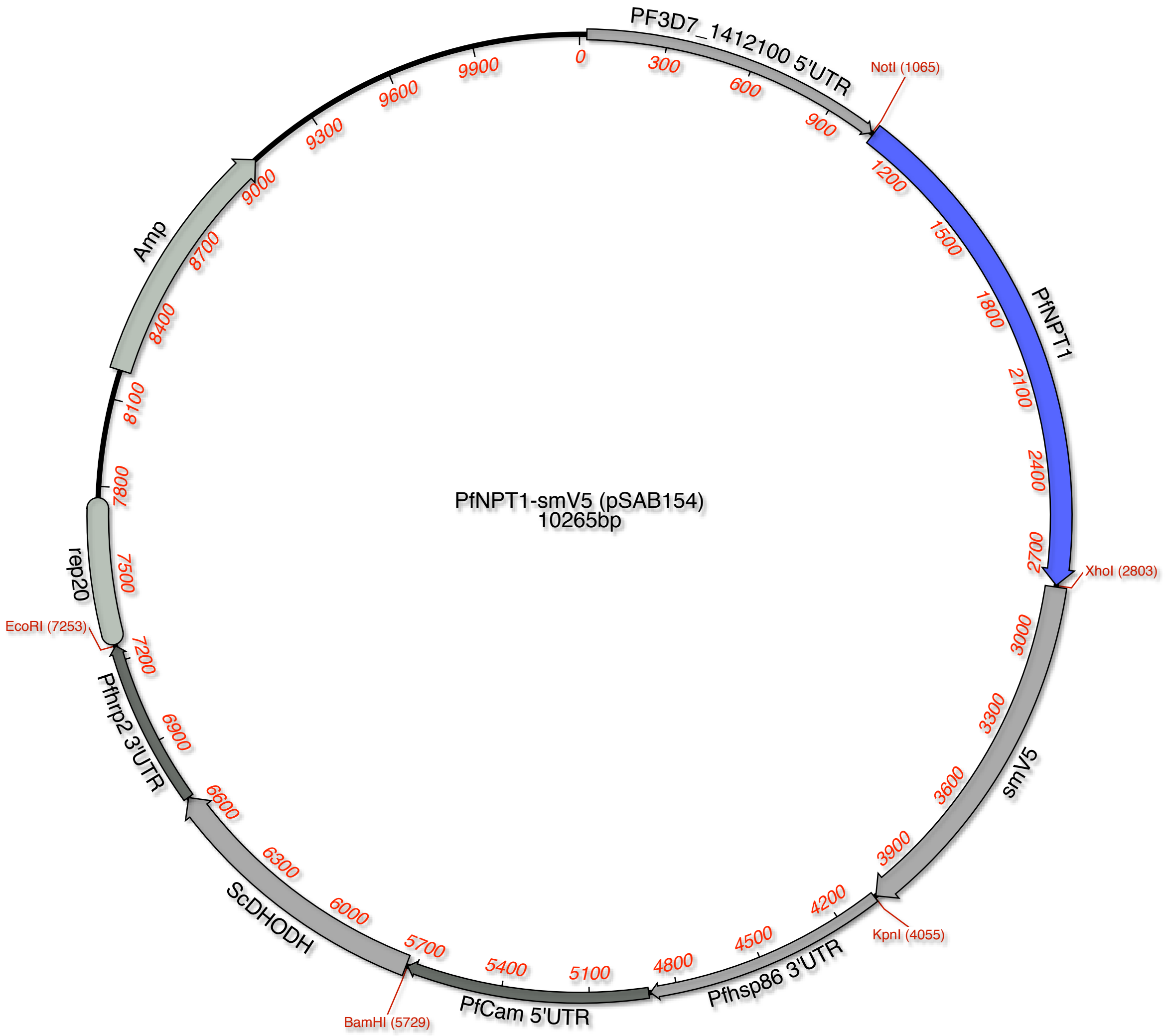

Figure S1. PfNPT-smV5 plasmid map. Plasmid map for episomal expression vector. The full-length coding region for PfNPT1 (PF3D7_0104800) was cloned downstream of the PF3D7_1412100 5'UTR. The resulting protein is a fusion between PfNPT1 and the spaghetti monster V5 epitope tag (smV5). The positive selection cassette expresses the dihydroorate dehydrogenase protein from *Saccharomyces cerevisiae* (ScDHODH).
